# Nutritional literacy and its influence on healthy lifestyle behavior and willingness to consume healthy food brands

**DOI:** 10.3389/fnut.2025.1680436

**Published:** 2025-11-25

**Authors:** Elizabeth Emperatriz García-Salirrosas, Manuel Escobar-Farfán, Iván Veas-González, Ledy Gomez-Bayona, Jorge Alberto Esponda-Perez, Ghenkis Ezcurra-Zavaleta, Elena Matilde Urraca-Vergara

**Affiliations:** 1 Grupo de Investigación e Innovación para el Emprendimiento y Sostenibilidad, Universidad Nacional Tecnológica de Lima Sur, Lima, Peru; 2 Department of Administration, Faculty of Administration and Economics, Universidad de Santiago de Chile (USACH), Santiago, Chile; 3 Departamento de Administración, Facultad de Economía y Administración, Universidad Católica del Norte, Antofagasta, Chile; 4 Facultad Ciencias Económicas Administrativas, Instituto Tecnológico Metropolitano, Medellin, Colombia; 5 Faculty of Nutrition and Food Sciences, Universidad de Ciencias y Artes de Chiapas, Tuxtla Gutiérrez, Mexico; 6 Facultad de Ciencias Económicas, Escuela de Administración, Universidad Nacional de Tumbes, Tumbes, Peru; 7 Programa de Estudio de Ingeniería Industrial, Facultad de Ingeniería, Universidad Privada Antenor Orrego, La Libertad, Peru

**Keywords:** nutritional literacy, healthy lifestyle behavior, healthy food brands, willingness to consume, nutritional marketing, Peruvian consumer

## Abstract

**Introduction:**

This study examines the influence of nutritional literacy on healthy lifestyle behavior and willingness to consume healthy food brands among Peruvian consumers of the Unión brand.

**Methods:**

Using structural equation analysis with data from 637 participants collected between June and September 2023.

**Results:**

The results confirm that nutritional literacy positively and significantly influences both the willingness to consume healthy brands (*β* = 0.367, *p* < 0.001)and healthy lifestyle behavior (*β* = 0.438, *p* < 0.001), while the latter also positively impacts the willingness to consume healthy brands (*β* = 0.220, *p* < 0.001). Additionally, the mediating effect of healthy lifestyle behavior on the relationship between nutritional literacy and willingness to consume healthy brands is verified (*β* = 0.096, *p* < 0.001).

**Discussion:**

These findings demonstrate that improving consumers’ nutritional knowledge not only directly influences their consumption preferences but also enhances this effect through the adoption of healthy habits. This offers relevant implications for marketing strategies focused on nutrition education.

## 1 Introduction

In recent decades, the global food landscape has undergone significant transformations, intensifying concerns about nutritional health and consumer well-being. The growing recognition of the relationship between diet and non-communicable chronic diseases, such as obesity, diabetes, and cardiovascular diseases, has positioned nutritional education as a critical factor for developing healthier societies (1–3). In today’s world, nutritional literacy has become an essential skill that enables people to successfully navigate the complex landscape of food information they encounter daily. This ability protects against diet-related diseases while encouraging the adoption of healthy and sustainable eating habits (3, 4).

It can be understood as the multifaceted ability to access, understand, and effectively apply nutritional information when making food choices. This concept has gained particular significance across Latin America, where dietary consumption patterns are undergoing rapid nutritional transitions (5–7). Nutritional literacy has enabled some households in Peru to adopt healthy eating habits (8). The arguments presented in some nutritious food sales promote greater awareness and modification in consumption habits, which foster better eating routines that align with exercise and the overall well-being of the individual. However, specifically in Peru, the Ministry of Health says that more than 60% of the population is overweight (8) and has health-related problems due to high levels of carbohydrate and sugar intake. Similarly, young people between 18 and 30 years old often lack healthy foods in their diets, and the opportunity to modify their diet to include less processed foods is limited.

In the Peruvian market, this issue assumes particular significance due to the country’s rich food cultural diversity and urbanization processes, which have altered consumption habits, especially among young urban populations. Food sector companies, such as the Unión brand, face the dual challenge of responding to growing demands for healthier products while actively contributing to consumer nutritional education. Contemporary consumer behavior strongly favors foods that contribute to health and well-being, driven by consumers’ enhanced access to tools that empower informed decisions about their nutritional choices (9, 10). Despite growing academic and commercial interest in these topics, a notable gap exists in the scientific literature regarding a comprehensive understanding of how nutritional literacy influences consumer behavior in Latin American contexts, most previous studies have focused on the direct relationship between nutritional knowledge and food choice, without sufficiently exploring the mediating role that general healthy lifestyle behaviors may play in this relationship. Furthermore, research in emerging markets such as Peru has been limited, representing a significant opportunity to contribute to academic knowledge while generating practical insights for the local food industry.

This research addresses a significant gap by investigating the impact of nutritional literacy on healthy lifestyle behaviors and individuals’ willingness to purchase healthy brand foods, with a specific focus on Peruvian consumers who utilize Unión brand products. Our study takes a multidimensional perspective, recognizing that nutritional literacy is a complex concept made up of both informational skills and expert competencies (11), while exploring both direct effects and mediation mechanisms through healthy lifestyle behavior. For the Peruvian context, adolescents prefer to consume sugary drinks to healthy foods; drinks and fast foods have marked a consumption behavior (12) that generates new opportunities for entrepreneurs in the diversification of the portfolio of products rich in carbohydrates, fried foods, and sauces that do not benefit the health of the population.

The primary goal of our research is to examine the structural relationships between nutritional literacy, healthy lifestyle behaviors, and willingness to consume healthy brand foods. We are particularly interested in understanding how healthy lifestyle behaviors might mediate the relationship between nutritional literacy and preferences for healthy brand consumption. This framework helps us better understand how nutritional knowledge translates into specific buying behaviors, especially since improved nutritional literacy gives consumers the cognitive tools they need to assess brand health claims, understand nutritional labels, and make well-informed food purchasing decisions (13–15).

What makes this research especially relevant is today’s environment, where consumers, particularly young urban populations, are constantly exposed to conflicting nutritional information. These consumers seek brands that meet their immediate food needs and match their long-term health and wellness values. In this sense, understanding how nutritional literacy shapes both healthy lifestyle behaviors and brand preferences becomes both an academic and commercial imperative that can significantly contribute to social well-being and sustainable development of the Peruvian food sector, especially considering that strategic marketing interventions, particularly nudge marketing tools, are advisable to promote consumers’ willingness to choose and consume healthier brand foods (11, 13).

## 2 Theoretical background

### 2.1 Theoretical foundations

This section presents the theoretical foundations underlying the proposed model, integrating four complementary perspectives: cognitive-behavioral frameworks, consistency theories, consumer behavior theory, and theories of behavioral change.

#### 2.1.1 Cognitive-behavioral frameworks: knowledge as a driver of behavior

Cognitive-behavioral frameworks posit that cognitive processes—including knowledge acquisition and information processing—serve as essential precursors to behavioral change (6, 7). In nutritional contexts, these frameworks suggest enhanced literacy provides cognitive tools for informed decision-making through a knowledge-to-action pathway. The mechanism operates through three processes: informational skills enable access to and comprehension of nutritional data; expert competencies allow for sophisticated interpretation in real-world contexts; and critical evaluation abilities facilitate the assessment of conflicting information (7). Educational interventions targeting nutritional literacy have proven effective in promoting healthy lifestyle adoption across diverse populations (16–18).

#### 2.1.2 Consistency theories: alignment between lifestyle and consumption

Consistency theories propose that individuals maintain coherence between beliefs, attitudes, values, and behaviors (19, 20). Inconsistency creates psychological discomfort, motivating the resolution of discrepancies. In consumer contexts, these theories explain why individuals strive for alignment between their lifestyle choices and consumption patterns. When consumers adopt healthy lifestyles, they develop expectations for coherence between behaviors and consumption choices (21, 22). The mechanism operates through three pathways: cognitive consistency drives the seeking of products that reinforce lifestyle identity; value alignment creates elevated quality expectations (23, 24); and consumption choices serve as visible demonstrations of lifestyle identity, expressing health commitments. Research demonstrates lifestyle behaviors significantly influence attitudes toward health-related products (20, 21).

#### 2.1.3 Consumer behavior theory: information processing in purchase decisions

Consumer behavior theory emphasizes that choices result from systematic information processing, evaluation, and decision-making (13, 25, 26). In healthy food contexts, nutritional literacy equips consumers with enhanced evaluative capabilities influencing willingness to engage with healthy brands. The mechanism operates through three pathways: nutritional literacy enables practical interpretation of labels and health claims (26); expert competencies allow navigation of complex marketing environments (25); and literacy reduces cognitive burden by providing structured evaluation frameworks (13). Research demonstrates brand healthiness perceptions, product quality, and clear nutritional information significantly impact willingness to purchase healthy branded foods (15, 26, 27).

#### 2.1.4 Behavioral change theories: sequential pathways to action

Behavioral change theories emphasize that change follows predictable stages, with knowledge and attitudes serving as precursors to behavioral adoption, which in turn reinforces future intentions (14). These theories provide a rationale for understanding mediation mechanisms, suggesting that nutritional literacy’s influence on consumption willingness operates both directly and indirectly through lifestyle behaviors. The mechanism involves three stages: knowledge acquisition creates cognitive readiness, cognitive foundation translates into behavioral change, and established behaviors create motivational contexts that influence consumption decisions.

### 2.2 Conceptualization of key constructs

#### 2.2.1 Nutritional literacy: a multidimensional construct

Nutritional literacy is a multifaceted construct that encompasses an individual’s capacity to effectively access, comprehend, and apply nutritional information in dietary decisions (5, 6). It involves gaining knowledge about the principles of healthy eating, interpreting nutritional information in foods, and making informed decisions about diet and nutrition (1, 7). Current research views nutritional literacy as a multidimensional concept that includes two main components: informational skills and expert competencies (11, 28). Informational skills involve our cognitive ability to find, critically evaluate, and effectively use nutritional information when making decisions. These skills serve as the foundation that helps people make well-informed choices about their diet and overall health management (28, 29). Meanwhile, expert competencies represent a more sophisticated level of nutritional understanding beyond basic information processing. This advanced level includes comprehensive knowledge of scientific dietary principles and the ability to apply evidence-based nutritional concepts in everyday situations. People with these competencies can contribute meaningfully to nutritional discussions, offer evidence-based advice to others, and navigate complex dietary decisions using their advanced nutritional knowledge (28, 30, 31).

Beyond these two primary dimensions, critical nutritional literacy represents an advanced skill level where people can skillfully evaluate and question nutrition information and advice. Those with critical nutritional literacy are also motivated to tackle nutritional challenges from individual, community, and global viewpoints (32). The importance of nutritional literacy in today’s society has increased dramatically, with growing interest and attention being paid to this area (2). Enhanced nutritional literacy protects against diet-related chronic diseases, including obesity and diabetes, while promoting sustainable healthy eating patterns and fostering healthy eating habits to make informed dietary decisions (1, 3).

#### 2.2.2 Healthy lifestyle behavior

Healthy lifestyle behavior encompasses the deliberate adoption of daily practices and habits that regulate an individual’s behavior regarding their contribution to their physical, mental, and social well-being (33, 34). This construct represents the decision to adopt habits and structure daily activities to help prevent diseases, increase longevity, improve quality of life, and increase life expectancy (35, 36).

The theoretical understanding of healthy lifestyle behavior is grounded in the recognition that every action associated with an individual’s daily behavior can have a significant impact on their physical, mental, and/or social health (19, 20, 22). These actions are referred to as lifestyles, as they form part of a pattern that influences the perceived quality of life. How individuals value their well-being influences their expectations, standards, and concerns (37). The quality of lifestyle choices directly correlates with perceived quality of life, where it is known that perceived quality can be evaluated according to the conduct of an individual’s lifestyle, and an unhealthy lifestyle minimizes the value of the quality of a particular product due to the behaviors and habits that affect their perception regarding the attributes that a product could offer (38).

Research demonstrates that lifestyle behaviors significantly influence consumer attitudes toward health-related products (22). A young person with a sedentary lifestyle may not prioritize the nutritional quality of a product because they do not perceive an immediate need for a healthy product that improves their health condition (39, 40). Conversely, when people adopt a healthy lifestyle, they tend to maintain high expectations when choosing a product and often opt for healthy brands as a food safety measure (23, 24). The construct encompasses various dimensions, including physical activity patterns, dietary choices, stress management practices, sleep hygiene, and preventive health behaviors. These interconnected components work synergistically to create holistic lifestyle patterns that support optimal health outcomes and influence consumer behavior in health-related contexts.

#### 2.2.3 Willingness to consume healthy brand foods

Willingness to consume healthy brand foods describes how ready consumers are to choose and eat branded foods they view as healthy or beneficial for their well-being (41–43). This concept captures the likelihood that someone will follow through on a behavior based on their motivations, beliefs, and perspectives about a specific product (44). Today’s consumers show a strong preference for foods that support their health and well-being, mainly because they now have better access to tools that help them make informed nutritional decisions (9, 10). This behavioral pattern also depends on the trust that products have built in the marketplace, especially since modern consumers generally have positive attitudes toward healthy foods (21, 45–47).

Several factors can influence someone’s attitude toward choosing these foods, including how they perceive the brand, the nutritional information available, price considerations, and their personal preferences (20, 48, 49). Research has shown that consumers’ willingness to buy and eat healthy branded foods is shaped by how healthy they think the brand is, their perception of product quality, and whether nutritional information is presented in an understandable way (15, 25–27). Socioeconomic factors also play a role—things like income level, education, and occupation can all affect people’s willingness to purchase branded healthy foods (21, 50, 51). To encourage consumers to choose and eat healthier brand foods, strategic marketing approaches are recommended, particularly nudge marketing techniques (52–54).

### 2.3 Conceptual model and research hypothesis

**Figure 1:**
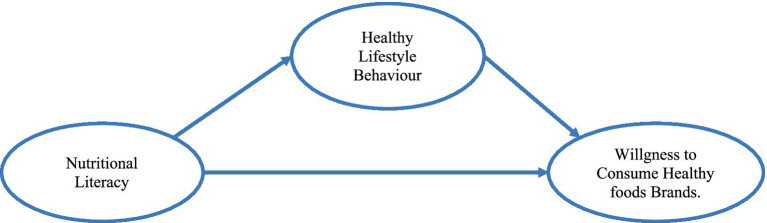
Conceptual model.

The proposed theoretical model (Figure 1) integrates the theoretical foundations discussed in Section 2.1, viewing nutritional literacy as a multidimensional independent variable that encompasses informational skills and expert competencies. This model suggests that nutritional literacy influences consumer behavior in two ways: directly and indirectly through its impact on healthy lifestyle behaviors. The framework combines cognitive-behavioral theory, consistency theory, and consumer behavior theory to explain the complex relationships between nutritional knowledge, lifestyle choices, and brand product choices.

#### 2.3.1 Influence of nutritional literacy on willingness to consume healthy brand foods

The connection between nutritional literacy and healthy lifestyle behavior is theoretically grounded in cognitive-behavioral frameworks (Section 2.1.1) that emphasize the role of knowledge and understanding in shaping behavioral patterns (6, 7). When people develop better nutritional literacy, they gain the cognitive tools needed to make informed dietary choices, which then influences their broader lifestyle behaviors (6, 7). People with higher nutritional literacy demonstrate superior ability in interpreting nutritional information, evaluating food options, and turning this knowledge into practical eating habits (1, 5). This deeper understanding creates a foundation for comprehensive healthy lifestyle patterns that extend beyond just food choices to include physical activity, stress management, and overall wellness behaviors.

The critical thinking skills associated with advanced nutritional literacy help people question nutritional claims, evaluate conflicting information, and make decisions that align with their health goals (32). This analytical ability supports the development of systematic healthy lifestyle behaviors by providing the foundation needed to make consistent, informed choices across different health-related areas. Educational programs focused on improving nutritional literacy have successfully encouraged healthy lifestyle adoption among diverse groups of people in various settings (16–18).

H2: Nutritional literacy positively influences healthy lifestyle behavior.

#### 2.3.2 Influence of nutritional literacy on healthy lifestyle behavior

The direct relationship between nutritional literacy and willingness to consume healthy brand foods is supported by consumer behavior theories that emphasize the importance of knowledge and understanding in purchase decision-making. Enhanced nutritional literacy equips consumers with the cognitive capabilities to evaluate brand health claims, interpret nutritional labels, and make informed decisions about food purchases (13, 25, 26). Consumers with higher nutritional literacy demonstrate superior ability to assess brand positioning, evaluate nutritional transparency, and distinguish between authentic health benefits and marketing claims (13, 25, 26). This enhanced evaluative capacity increases their willingness to engage with brands that provide genuine nutritional value and transparent health information.

The informational and expert competencies associated with nutritional literacy enable consumers to navigate complex food marketing environments and identify brands that align with their health goals (3, 14). This capability reduces uncertainty in food choices and increases confidence in selecting healthy branded products. Clear and accessible nutritional labeling, promoted through enhanced literacy programs, facilitates consumer understanding and supports informed brand selection (4, 10, 27, 55). Marketing strategies emphasizing nutritional transparency and comprehensive health information are more effective with nutritionally literate consumers who can appreciate and utilize this information.

H1: Nutritional literacy positively influences willingness to consume healthy brand foods.

#### 2.3.3 Influence of healthy lifestyle behavior on willingness to consume healthy brand foods

The relationship between healthy lifestyle behavior and willingness to consume healthy brand foods is theoretically supported by consistency theories, which suggest that individuals seek coherence between their lifestyle choices and consumption behaviors. Consumers who maintain healthy lifestyles develop elevated expectations for product quality and demonstrate preferences for brands that align with their wellness goals (23, 24). Healthy lifestyle behaviors create psychological and practical contexts that favor health-oriented consumption choices. Individuals engaged in regular physical activity, balanced nutrition, and wellness practices develop heightened awareness of the relationship between consumption choices and health outcomes (23, 24). This awareness translates into increased willingness to invest in healthy branded foods as part of their comprehensive health maintenance strategy.

The pursuit of healthy lifestyle behaviors creates motivational frameworks that support health-conscious consumption patterns. Consumers committed to wellness goals seek products and brands that reinforce and support their lifestyle choices, increasing their willingness to purchase healthy branded foods (33, 34, 56, 57). Research demonstrates that lifestyle quality influences consumer perceptions of product value, with healthy lifestyle adopters placing greater importance on nutritional quality and health benefits (20, 38, 56). This heightened valuation increases their willingness to consume and invest in healthy branded products that support their lifestyle goals.

H3: Healthy lifestyle behavior positively influences willingness to consume healthy brand foods.

#### 2.3.4 Mediating role of healthy lifestyle behavior

The mediating role of healthy lifestyle behavior in the relationship between nutritional literacy and willingness to consume healthy brand foods represents a key theoretical contribution of this model. This mediation relationship suggests that nutritional literacy influences brand consumption willingness directly and indirectly through its impact on lifestyle behavior patterns (Section 2.1.4) (14). Nutritional literacy provides the cognitive foundation for making informed health decisions, which subsequently leads to adopting healthy lifestyle behaviors (4, 6). These lifestyle behaviors create motivational and practical contexts that increase the willingness to consume healthy branded foods, establishing a pathway through which nutritional knowledge translates into consumption behavior. Behavioral change theories support the mediation model, emphasizing the sequential nature of attitude-behavior relationships. Enhanced nutritional literacy influences health behaviors and lifestyle patterns, creating specific consumption preferences and brand selection criteria (14). This mediating relationship suggests that interventions targeting nutritional literacy may impact consumer behavior more than previously recognized. They influence direct food choices and comprehensive lifestyle patterns, shaping brand preferences and willingness to consume.

H4: Healthy lifestyle behavior mediates the relationship between nutritional literacy and willingness to consume healthy brand foods.

## 3 Materials and methods

### 3.1 Sample

This study employed a quantitative cross-sectional design to investigate the relationships between nutritional literacy, healthy lifestyle behavior, and willingness to consume healthy food brands. The population consisted of Peruvian adult consumers of Unión brand products, chosen explicitly because Unión is one of the most recognized and widely distributed healthy food brands in Peru (58–61). Its market relevance and strong positioning in nutritional education campaigns provided a suitable context for analyzing consumer behavior in emerging markets.

Participants were recruited using non-probability convenience sampling, a methodologically appropriate approach when accessibility and contextual relevance are paramount considerations in consumer research (62). Inclusion criteria were: being over 18 years of age, residing in Lima, Peru, and having consumed at least one Unión product in the 6 months before the survey. The final sample comprised 637 respondents, exceeding the recommended minimum of 10 observations per item in structural equation modelling (63). With 23 items across the three constructs, the minimum requirement was 230 participants; thus, the achieved sample size exceeded the threshold, ensuring robust statistical power.

The sample was predominantly female (65.1%) and young people aged 18–25 (85.4%). Most were single (93.4%) and had an undergraduate university education (89.5%). Regarding income, 52.9% reported earning up to two minimum monthly salaries. The details are summarized in Table 1, which reflects a homogeneous profile targeting urban youth with access to higher education.

**Table 1:** Sociodemographic data.

Sociodemographic variable	Category	Frequency	Percentage
Sex	Man	222	34.90%
	Woman	415	65.10%
Age	18–25 years old	544	85.40%
	26–33 years old	57	8.90%
	34–41 years old	20	3.10%
	Older than 41 years old	16	2.50%
Marital status	Married	35	5.50%
	Cohabiting	2	0.30%
	Divorced	5	0.80%
	Single	595	93.40%
Academic level	College	19	3.00%
	Advanced technical degree	7	1.10%
	University (postgraduate)	41	6.40%
	University (undergraduate)	570	89.50%
Income	From 11 to 20 minimum wages	26	4.10%
	From 3 to 4 minimum wages	155	24.30%
	From 5 to 10 minimum wages	105	16.50%
	Up to 2 minimum wages	337	52.90%
	More than 20 minimum wages	14	2.20%

Ethical considerations were guaranteed by obtaining digital informed consent, ensuring voluntary participation, anonymity in data management, and exclusive use for academic purposes, following the guidelines of the Declaration of Helsinki (60). Sociodemographic data were collected without disclosing personal information, in accordance with confidentiality regulations, and to protect the integrity of the participants.

### 3.2 Measurements

The instrument included validated scales for nutritional literacy (11), healthy lifestyle behavior (61), and willingness to consume healthy brands (64), all presented on a 5-point Likert scale ranging from “strongly disagree” to “strongly agree,” adjusted to the Peruvian context. Data collection took place between June and September 2023, covering various urban regions in the country.

### 3.3 Statistical analysis

To verify validity and reliability, internal consistency was assessed using Cronbach’s alpha (>0.70) and composite reliability (>0.70), convergent validity was confirmed with AVE (>0.50), and discriminant validity was tested using the HTMT criterion (<0.90) (65). The data did not follow a normal distribution; however, the PLS-SEM model is robust enough to handle this type of non-normal data (66). The statistical analysis was conducted using SmartPLS 4. Bootstrapping with 5,000 subsamples was applied to evaluate path significance (*p* < 0.05). Model quality was assessed using *R*^2^ for explanatory power.

## 4 Results

Table 2 presents the validation of the final measurement model, focusing on the reliability and convergent validity of the study’s three primary constructs: For the healthy lifestyle behavior (HLB) variable, the five outer loadings of its indicators (HLB1–HLB5) present factor loadings ranging from 0.695 to 0.801, exceeding the recommended threshold of 0.7 (with HLB1 slightly below but acceptable), indicating a good correlation between the indicators and the construct. Cronbach’s alpha reached 0.829, above the 0.7 threshold, demonstrating high internal consistency among the indicators. The composite reliability (rho_a) was 0.829, above 0.7, indicating good reliability. The Average Variance Extracted (AVE) was 0.66, above the 0.5 threshold, indicating that the construct explains more than 66% of the variance of its indicators.

**Table 2:** Validation of the first and second order measurement model with reliability and convergent validity.

Variable	Code	Outer loadings	Cronbach’s alpha	Composite reliability (rho_c)	Average variance extracted
First order					
Healthy lifestyle behavior (HLB)	HLB1	0.696	0.794	0.798	0.549
	HLB2	0.801			
	HLB3	0.751			
	HLB4	0.726			
	HLB5	0.724			
Information skills (nutritional literacy)	NL1(IS)	0.736	0.895	0.897	0.614
	NL2(IS)	0.827			
	NL3(IS)	0.801			
	NL4(IS)	0.827			
	NL5(IS)	0.731			
	NL6(IS)	0.793			
	NL8(IS)	0.767			
Expert skills (nutritional literacy)	NL7(ES)	0.753	0.829	0.829	0.661
	NL9(ES)	0.834			
	NL10(ES)	0.826			
	NL11(ES)	0.837			
Willingness to consume healthy food brands (WCHFB)	WCHFB1	0.823	0.928	0.930	0.698
	WCHFB2	0.847			
	WCHFB3	0.810			
	WCHFB4	0.851			
	WCHFB5	0.869			
	WCHFB6	0.840			
	WCHFB7	0.807			
Second order					
Nutritional literacy	IS EX	0.923 0.895	0.790	0.858	0.826

For the variable nutritional literacy (NL), which is composed of two reflective subdimensions—information skills and expert skills—the outer loadings of the eleven items (NL1–NL11) ranged from 0.731 to 0.831, all exceeding the 0.7 threshold, indicating a strong relationship with the construct. The Information Skills subdimension was measured using items NL1 to NL6, as well as NL8. Expert Skills, on the other hand, included items NL9 to NL11, with NL7 also taken into account. The Information Skills subdimension demonstrated excellent internal consistency, with a Cronbach’s alpha of 0.895, which is well above the recommended threshold of 0.7. The composite reliability was 0.897, also well above 0.7, indicating high reliability. Additionally, the average variance extracted (AVE) was 0.614, surpassing the 0.5 criterion. In turn, the Expert Skills subdimension also demonstrated strong internal consistency, with a Cronbach’s alpha of 0.829, a composite reliability of 0.829, and an AVE of 0.661, all of which exceeded the recommended thresholds. Since the model considers nutritional literacy as a second-order reflective–reflective construct, composed of the subdimensions information skills and expert skills, the transformation from first-order to second-order constructs was carried out following the two-step approach proposed by Wright et al. (69). The results obtained for the second-order construct demonstrated adequate levels of reliability and validity, as shown in Table 2.

Regarding willingness to consume healthy food brands (WCHFB), the outer loadings of the seven indicators (WCHFB1–WCHFB7) range from 0.807 to 0.868, all of which are clearly above 0.7, demonstrating a strong association with the construct. Cronbach’s alpha was 0.928, well above 0.7, indicating excellent internal consistency. The composite reliability was 0.930, substantially exceeding the minimum threshold of 0.7, indicating a high level of composite reliability. The AVE was 0.698, significantly above 0.5, indicating that the construct explains nearly 70% of the variance in its indicators.

Table 2 confirms that the measurement model adequately satisfies the validity and reliability criteria required in research of this type. All constructs demonstrate excellent internal consistency (Cronbach’s alpha >0.82), high composite reliability (values >0.82), and good convergent validity (AVE > 0.55). Additionally, the indicators are adequately correlated with their respective constructs. These results statistically validate the robustness of the scales used to measure the three primary constructs of the study, providing a solid foundation for analyzing the structural relationships among them.

Discriminant validity was assessed using the heterotrait-monotrait (HTMT) ratio, following the recommendations of Henseler et al. (65), as it is considered one of the most robust and accurate methods to determine whether constructs are conceptually distinct. Table 3 presents the HTMT values among the first-order constructs: healthy lifestyle behavior (HLB), information skills (IS), expert skills (ES), and willingness to consume healthy food brands (WCHFB). All HTMT values were below the conservative threshold of 0.85, thus supporting adequate discriminant validity at the first-order level. Notably, the HTMT value between the subdimensions of Nutritional Literacy, IS, and ES was 0.848—close to the threshold but still acceptable given their conceptual proximity as components of the same higher-order construct. In addition, the HTMT values between HLB and IS (0.485), HLB and ES (0.510), IS and WCHFB (0.491), HLB and WCHFB (0.435), and ES and WCHFB (0.458) all remained below the threshold, confirming that these constructs represent theoretically distinct dimensions.

**Table 3:** Discriminant validity first-order heterotrait-monotrait ratio (HTMT)—matrix.

Variables	HLB	IS	ES	WCHFB
Healthy lifestyle behavior (HLB)				
Informational skills (NL)	0.485			
Expert skills (NL)	0.510	0.848		
Willingness to consume healthy food brands (WCHFB)	0.435	0.491	0.458	

Subsequently, the discriminant validity of the second-order construct Nutritional Literacy (NL), composed of the IS and ES subdimensions, was assessed using the two-step approach proposed by Wright et al. (2012). As shown in Table 4, the HTMT values between this second-order construct and the other key variables of the model also remained well below the 0.85 cut-off. Specifically, the HTMT value between NL and HLB was 0.489, and between NL and WCHFB it was 0.427. These results reaffirm that Nutritional Literacy, conceptualized as a second-order construct, is distinct from both healthy lifestyle behavior and willingness to consume healthy food brands.

**Table 4:** Discriminant validity second-order heterotrait-monotrait ratio (HTMT)—matrix.

Variables	HLB	NL	WCHFB
Healthy lifestyle behavior (HLB)			
Nutritional literacy (NL)	0.489		
Willingness to consume healthy food brands (WCHFB)	0.435	0.427	

Taken together, the findings reported in Tables 3, 4 provide strong evidence of discriminant validity at both the first- and second-order levels. This ensures that the model’s latent constructs capture non-overlapping theoretical concepts, thereby enhancing the validity of the structural relationships and reinforcing the methodological robustness of the study.

**Figure 2:**
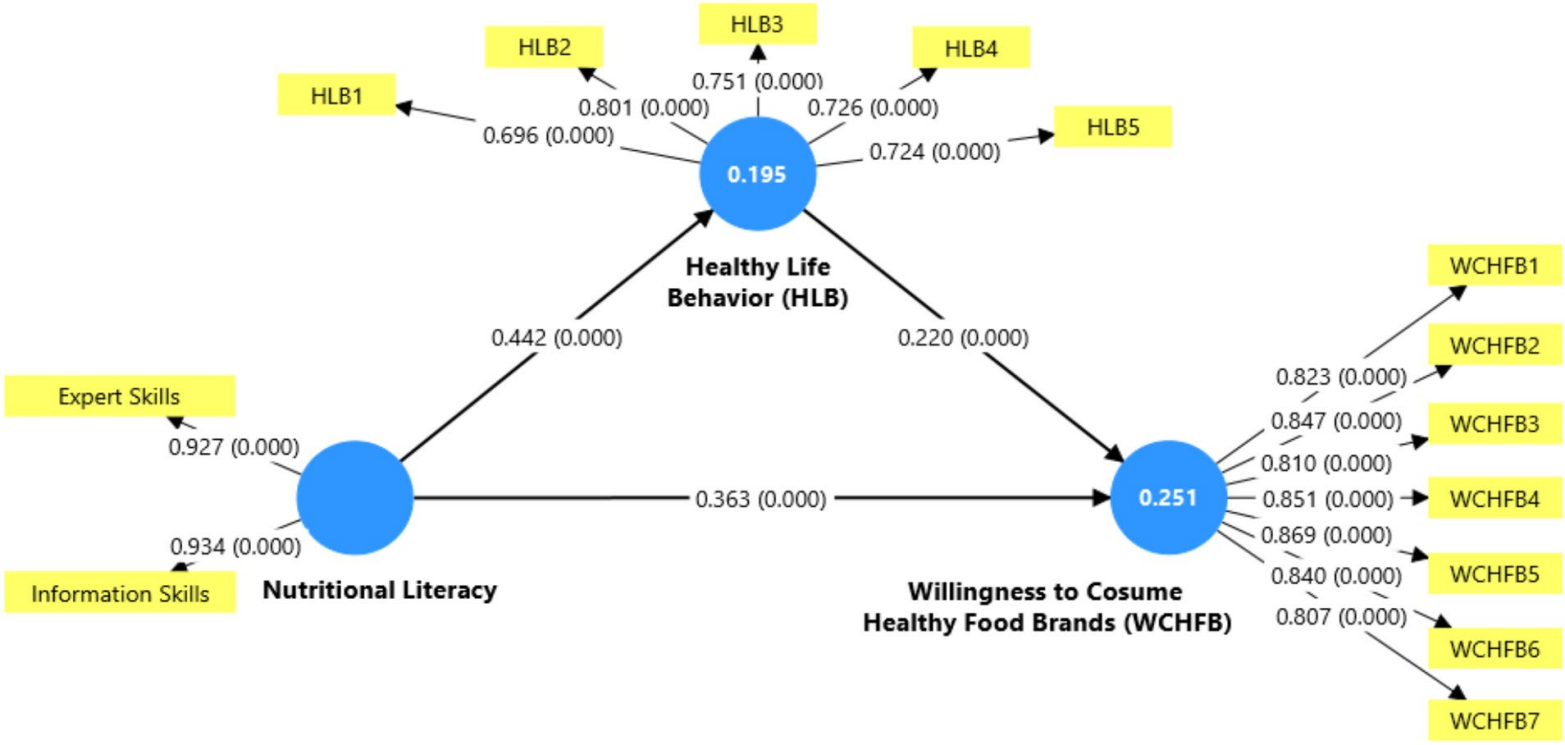
Structural model.

Figure 2 presents the structural model with the study’s statistical results on the relationship between nutritional literacy, healthy lifestyle behavior, and willingness to consume healthy food brands. Regarding the proposed relationships, all hypotheses are confirmed with statistically significant values (*p* < 0.000): nutritional literacy positively and significantly influences willingness to consume healthy food brands (WCHFB) with a coefficient of 0.363 (H1), explaining approximately 25.1% of its variance (as indicated by the 0.251 value in the WCHFB circle). Nutritional literacy has a positive and significant effect on healthy lifestyle behavior (HLB) with a coefficient of 0.442 (H2), explaining approximately 19.5% of its variance (as shown by the 0.195 value in the HLB circle). Healthy lifestyle behavior has a positive and significant influence on the willingness to consume healthy food brands, with a coefficient of 0.220 (H3). The mediating effect of healthy lifestyle behavior in the relationship between nutritional literacy and willingness to consume healthy brands is confirmed (H4, not explicitly shown in the coefficients but implicit in the model structure). Additionally, the model shows that the indicators of each construct have high and significant factor loadings. The healthy lifestyle behavior indicators (HLB1–HLB5) present values between 0.696 and 0.801, while the willingness to consume healthy food brand indicators (WCHFB1–WCHFB7) show values between 0.807 and 0.869, all statistically significant (*p* < 0.000). These results confirm that nutritional literacy, through its informational and expert skills components, is a substantial determinant of healthy lifestyle behavior and willingness to consume healthy food brands. Healthy lifestyle behavior also acts as an essential mediator in this relationship.

Table 5 presents the results of the structural equation analysis, confirming all proposed hypotheses in the theoretical model with high statistical significance (*p* < 0.001). Nutritional literacy has a direct and significant influence on the willingness to consume healthy food brands (*β* = 0.363, *t* = 8.789) and healthy lifestyle behaviors (*β* = 0.442, *t* = 10.531). Similarly, healthy lifestyle behavior has a positive influence on the willingness to consume healthy food brands (*β* = 0.220, *t* = 5.170), and the significant mediating effect of healthy lifestyle behavior in the relationship between nutritional literacy and willingness to consume healthy brands is verified (*β* = 0.097, *t* = 4.696). These findings demonstrate that nutritional literacy is a fundamental determinant of healthy food consumption preferences, both directly and indirectly, through promoting healthy lifestyle behaviors. This suggests the importance of strengthening nutritional education to promote healthier consumption patterns in the food market.

**Table 5:** Hypothesis testing.

H	Relationships	Original sample (O)	Sample mean (M)	Standard deviation (STDEV)	T statistics (|O/STDEV|)	*p* values	Decision
H1	Nutritional_Literacy → Willingness to Consume_Healthy Food Brands_(WCHFB)	0.363	0.365	0.041	8.789	0.000	Accepted
H2	Nutritional_Literacy → Healthy Lifestyle _Behavior_(HLB)	0.442	0.443	0.042	10.531	0.000	Accepted
H3	Healthy Lifestyle _Behavior_(HLB) → Willingness to Consume_Healthy Food Brands_(WCHFB)	0.220	0.221	0.043	5.170	0.000	Accepted
H4	Nutritional _Literacy → Healthy Lifestyle_Behavior_(HLB) → Willingness to Consume_Healthy Food Brands_(WCHFB)	0.097	0.098	0.021	4.696	0.000	Accepted

## 5 Discussion

This study confirms that nutritional literacy is a significant and determining factor in shaping decisions regarding healthy eating habits. The results indicate that individuals with a greater capacity to understand and interpret nutritional information tend to adopt healthier lifestyles and are more willing to consume food brands perceived as beneficial to their health.

The findings not only confirm the direct effect of nutritional literacy on both healthy lifestyle behavior and willingness to consume healthy food brands but also validate the mediating role of lifestyle behaviors. This result contributes to cognitive-behavioral and consistency theories by demonstrating that consumer choices are not driven solely by informational capacities but also by the alignment between knowledge and daily practices (7, 19). In this sense, the study provides empirical evidence that nutritional literacy creates a foundation for behavior change that is reinforced when individuals incorporate healthy routines into their lifestyles.

Compared with previous studies in European and North American contexts (29, 34), our results highlight that these mechanisms also operate in emerging markets such as Peru. This reinforces the universality of nutritional literacy as a determinant of health-related consumption, while also highlighting contextual nuances. Despite economic constraints, younger consumers prioritize brands that offer nutritional value, suggesting that health considerations are gradually becoming a stronger driver than price alone.

Theoretically, this study enriches the literature on nutritional literacy by modeling it as a multidimensional construct (information skills and expert competencies), thus providing a more refined measurement approach (8, 9). This perspective enables a deeper understanding of how various types of literacy impact health behaviors and brand preferences. In practice, the findings suggest that companies should invest in educational marketing strategies that go beyond promotional messages and actively contribute to strengthening consumer knowledge and skills. For policymakers, the evidence underscores the potential of nutritional literacy programs to promote healthier consumption patterns, especially among young populations.

Moreover, this direct and mediated relationship supports the findings of Vettori et al. (7) found that nutritional knowledge affects dietary choices and general health attitudes. Our research is distinctive because we have empirically validated that healthy lifestyle behavior serves as a mediator. In other words, nutritional literacy substantially affects purchasing decisions when people have internalized healthy habits into their daily lives. This complex relationship supports Guttersrud et al.’s. (32) approach, which advocates for developing critical literacy beyond simply reading labels, enabling consumers to compare, question, and make decisions based on their overall well-being. Our findings also align with cognitive-behavioral models, suggesting that knowledge alone does not change behavior unless it is accompanied by sustained motivation and practice. (6).

When we compare these results with studies from other contexts, we see consistency with Demartini et al.’s (13) findings, which showed that nutritional literacy influences the value people place on healthy brands, are crucial in markets where health considerations increasingly drive purchasing decisions—similarly, research by García-Salirrosas et al. (22) demonstrated that consumers with healthy habits place greater importance on brand transparency and reputation, demanding consistency between advertising messages and the actual nutritional value of products.

In the Peruvian context, focusing on a well-known brand like Unión allowed us to explore how these concepts play out in a specific environment characterized by young people with high levels of education but economic constraints. Despite these financial limitations, we confirmed that nutritional literacy has a significant influence on purchasing behavior, even in emerging market contexts. This supports Plasek et al.’s (15) findings, which noted that although price remains an essential factor, perceptions of nutritional value can shift preferences, particularly among younger consumers.

Another important aspect involves leveraging these findings from a business and public policy perspective. Brands can strengthen their positioning by investing in educational marketing strategies. At the same time, health programs can incorporate nutritional literacy components as tools for changing consumption patterns—studies such as those by Grunert et al. (55) and Priya and Alur (10) suggest that when nutritional information is clear and accessible, consumers understand better and trust brands more. Therefore, this research affirms that nutritional literacy drives change in consumer behavior and that its effect is amplified when accompanied by healthy habits. The willingness to consume nutritious foods does not respond solely to advertising campaigns but to a cognitive and practical process built into the consumer’s daily life. This process is also influenced by cultural, social, and educational factors, opening a broad research agenda for future studies in Latin America.

### 5.1 Theoretical and practical implications

Theoretically, this study makes a significant contribution to the knowledge of consumer behavior in the healthy food domain. The results empirically validate a conceptual model that integrates nutritional literacy, healthy lifestyle behavior, and willingness to consume healthy food brands, extending previous literature on nutritional marketing. The confirmed mediating effect of healthy lifestyle behavior enriches understanding of how nutritional literacy influences consumption decisions, suggesting that this process is more complex than a simple direct relationship. In practical terms, the findings provide valuable guidelines for food companies like Unión. Marketing strategies should focus on communicating product benefits and educating consumers about nutritional aspects, strengthening both their informational and expert skills. Corporate social responsibility programs could be oriented toward promoting nutritional literacy and healthy lifestyles, thus creating a dual benefit: improving public health and increasing preference for healthy food brands.

### 5.2 Limitations and future research

This study presents some limitations that should be considered when interpreting its results. First, the sample consisted primarily of university students (85.4% between 18 and 25 years old and 89.5% with an undergraduate university education), which limits the generalizability of the findings to other demographic segments. Second, by focusing exclusively on consumers of the Unión brand in Peru, the results might not be extrapolable to other brands or cultural contexts. Third, the study’s cross-sectional design does not allow for establishing definitive causal relationships between variables, as it does not capture the temporal evolution of these relationships. Finally, although the model presents good validity and reliability indicators, other unconsidered variables could influence the studied relationships. Additionally, certain methodological aspects require acknowledgment. First, the reliance on self-report questionnaires may introduce potential biases, such as social desirability or recall errors, which could influence the accuracy of responses (67). Second, the use of non-probability convenience sampling limits the representativeness of the sample, thereby constraining the generalizability of the findings to the broader Peruvian population (12). Third, the exclusive use of online surveys may have excluded individuals without stable internet access or with lower levels of digital literacy, potentially biasing the sample toward younger and more technologically engaged consumers (68). Addressing these limitations in future studies through probabilistic sampling strategies, mixed data collection methods, and complementary qualitative designs would enhance the robustness and external validity of the results.

Future research could address the mentioned limitations and expand knowledge in this field. Conducting longitudinal studies to evaluate the evolution of relationships between nutritional literacy, healthy lifestyle behaviors, and consumption preferences would be valuable. Expanding research to more diverse samples of age, educational level, and socioeconomic context would allow a better understanding of the model’s applicability across different population segments. It would also be interesting to include moderating variables such as cultural influences, social pressure, or economic accessibility to healthy foods. Comparative research between different brands or categories of nutritious food products could reveal whether the identified effects are generalizable or specific to particular market contexts. Ultimately, experimental studies that manipulate elements of nutritional literacy could provide more robust evidence of causal relationships.

## 6 Conclusion

This study presents several strengths that reinforce its contribution to the literature. First, it is based on a relatively large sample size (*n* = 637), which provides sufficient statistical power and precision in the estimates. Second, the constructs were measured using validated multidimensional scales, ensuring reliable operationalization of nutritional literacy, healthy lifestyle behaviors, and willingness to consume healthy food brands. Third, the application of structural equation modeling with the PLS-SEM technique, accompanied by robust validity and reliability assessments, brings methodological rigor to the study (12, 67). Ultimately, by focusing on an underexplored Latin American context, the research provides novel insights with both theoretical relevance and practical implications for marketing strategies and public health policies.

This study demonstrates that nutritional literacy, encompassing both informational and expert skills, is a significant determinant of healthy lifestyle behaviors and the willingness to consume healthy food brands. The results confirm that this influence operates both directly and indirectly, with healthy lifestyle behavior serving as an significant mediating factor in this relationship. These findings underscore the significance of nutritional education as a vital tool for enhancing public health and as a strategic factor for companies aiming to establish their brands in the expanding healthy food market. The empirical evidence presented suggests that strengthening consumers’ nutritional literacy can generate a virtuous cycle: Greater nutritional knowledge promotes healthier behaviors, which in turn reinforce preference for healthy food brands. This more profound understanding of the mechanisms that influence food consumption decisions provides valuable guidelines for policymakers seeking to improve population health and companies committed to promoting healthier food alternatives.

Overall, the evidence demonstrates that nutritional literacy is not only a determinant of healthy consumption but also a mechanism that operates indirectly through lifestyle habits, amplifying its effect on consumer choices. This dual pathway suggests that strategies that combine educational initiatives with behavioral reinforcement will be more effective than isolated interventions. For marketers, the results underscore the importance of transparent communication and nutritional education as key tools in fostering brand loyalty within the health food sector. For policymakers, the study reinforces the importance of integrating nutritional literacy into public health strategies as a long-term investment in population well-being. By addressing these dimensions simultaneously, both academics and practitioners can gain a deeper understanding of and influence the complex relationship between knowledge, behavior, and consumption in the context of healthy food brands.

## Data Availability

The original contributions presented in the study are included in the article/supplementary material, further inquiries can be directed to the corresponding authors.
